# Antimicrobial and Anti-Inflammatory Activity of Apple Polyphenol Phloretin on Respiratory Pathogens Associated With Chronic Obstructive Pulmonary Disease

**DOI:** 10.3389/fcimb.2021.652944

**Published:** 2021-11-22

**Authors:** Rahel L. Birru, Kiflai Bein, Natalya Bondarchuk, Heather Wells, Qiao Lin, Y. Peter Di, George D. Leikauf

**Affiliations:** Department of Environmental and Occupational Health, Graduate School of Public Health, University of Pittsburgh, Pittsburgh, PA, United States

**Keywords:** bacteria, phloretin, antibacterial, anti-inflammatory, neutrophil, COPD

## Abstract

Bacterial infections contribute to accelerated progression and severity of chronic obstructive pulmonary disease (COPD). Apples have been associated with reduced symptoms of COPD and disease development due to their polyphenolic content. We examined if phloretin, an apple polyphenol, could inhibit bacterial growth and inflammation induced by the main pathogens associated with COPD. Phloretin displayed bacteriostatic and anti-biofilm activity against nontypeable *Haemophilus influenzae* (NTHi), *Moraxella catarrhalis*, *Streptococcus pneumoniae*, and to a lesser extent, *Pseudomonas aeruginosa*. *In vitro*, phloretin inhibited NTHi adherence to NCI-H292 cells, a respiratory epithelial cell line. Phloretin also exhibited anti-inflammatory activity in COPD pathogen-induced RAW 264.7 macrophages and human bronchial epithelial cells derived from normal and COPD diseased lungs. In mice, NTHi bacterial load and chemokine (C-X-C motif) ligand 1 (CXCL1), a neutrophil chemoattractant, was attenuated by a diet supplemented with phloretin. Our data suggests that phloretin is a promising antimicrobial and anti-inflammatory nutraceutical for reducing bacterial-induced injury in COPD.

## Introduction

Exposure to oxidants formed during infection and environmental exposures, such as tobacco smoke, and the resulting inflammation are major factors in chronic respiratory disease development and progression (Birru and Di, 2012; Barnes, 2020). Diet is a potential factor that could influence respiratory health in response. Past cross-sectional epidemiological studies have found associations between diets rich in fruits and vegetables and protection of the airways against oxidant-mediated damage that leads to COPD (Smit et al., 1999; Tabak et al., 1999; Butland et al., 2000; Tabak et al., 2001). For example, an inverse relationship was observed between baseline consumption of total and solid (i.e. apples and pears) fruit and incidence of chronic lung disease over 25 years in a cohort of 793 men (Miedema et al., 1993). Similarly, daily apple intake has been associated with reduced incidence of cough and phlegm production in a population with chronic respiratory symptoms (Butler et al., 2004). A more recent study by Kaluza et al. found that high total fruit and vegetable consumption (≥5.3 servings/day) as compared with low consumption (<2 servings/day) was associated with a reduced risk of COPD by 40% in current smokers and by 34% in ex-smokers (Kaluza et al., 2017). In longitudinal studies that investigated the association of vitamin C intake with indicators of COPD, no clear association has been observed (Miedema et al., 1993; Butland et al., 2000; Walda et al., 2002). Thus, apple and other fruit polyphenols with antioxidant properties were noted as possible contributors to the beneficial effects found in these studies.

Apple polyphenols include quercetin glycoside, chlorogenic acid, epicatechin, and phloretin. A member of the chalcone family of 1,3-diaryl-2-propen-1-ones, phloretin could be of particular interest as an intervention in COPD due to its broad range of therapeutic targets (Behzad et al., 2017). Phloretin can inhibit the growth and biofilm formation of various bacteria by disrupting energy metabolism (Barreca et al., 2014), nuclear conjugated additions to essential bacterial proteins (Nowakowska, 2007), and suppressing fimbria production (Lee et al., 2011). Phloretin also can inhibit inflammatory mediator secretion induced by bacterial lipopolysaccharide (LPS) and cigarette smoke exposure by reducing activation of the mitogen-activated protein kinase (MAPK) pathway and p65, a component of nuclear transcription factor kappa-B (Huang et al., 2013; Huang et al., 2016; Wang et al., 2018).

In this study, we investigated the potential role of phloretin to protect against the primary respiratory pathogens isolated from persons with COPD. The COPD lung is more prone to airway infections due to impaired mucociliary clearance and lung defense (Sethi, 2010). Pathogenic bacteria accelerate disease progression, mucus overproduction, and lung function decline in COPD and increase frequency of acute exacerbations of COPD (AECOPD), which are sudden worsening of disease symptoms (Patel et al., 2002; Wilkinson et al., 2003; Sethi and Murphy, 2008). AECOPD are associated with irreversible declines in lung function. The primary pathogens isolated from persons experiencing AECOPD are nontypeable *Haemophilus influenzae* (NTHi), *Moraxella catarrhalis* (*M. catarrhalis*), *Streptococcus pneumoniae* (*S. pneumoniae*), and *Pseudomonas aeruginosa* (*P. aeruginosa*) (Sethi et al., 2002; Sethi and Murphy, 2008).

The antibacterial effects of phloretin have primarily been assessed in cell free systems. Limited *in vitro* and *in vivo* work has been performed examining the role of phloretin against pathogen-induced inflammation and bacterial growth. Previously, Lee et al. found that phloretin could reduce *Escherichia coli* O157:H7 adhesion to and TNF-α-induced monocyte adhesion to HT-29 human colonic epithelial cells (Lee et al., 2011). In addition, in a rat model of colitis induced by trinitrobenzene sulfonic acid (TNBS), phloretin significantly ameliorated colon inflammation and body weight loss (Lee et al., 2011). Similarly, phloretin inhibited *Listeria monocytogenes* bacterial burden and inflammatory lesions in the livers and spleens of mice and improved their survival (Wang et al., 2017). We examined the effects of phloretin on COPD associated bacterial growth and induced inflammation in cell coculture models and in mouse lungs. Furthermore, to more closely model the human exposure, we administered phloretin orally through supplemented chow. We demonstrate that dietary administration of phloretin is a promising therapeutic to inhibit injury induced by the predominant pathogens associated with COPD.

## Materials and Methods

### Materials

A complete list of the reagents, cells, and animals and their commercial sources is provided (**Supplemental Table 1**). Briefly, a clinical isolate of nontypeable *Haemophilus influenzae* (NTHi) was provided by Dr. Hong Wei Chu (National Jewish Health). *Moraxella catarrhalis* (*M. catarrhalis*), *Streptococcus pneumoniae* (*S. pneumoniae*), and *Pseudomonas aeruginosa* (*P. aeruginosa*) strain PAO1 were obtained from ATCC. LPS from *P. aeruginosa* was obtained from Sigma-Aldrich. Chocolate agar and sheep blood agar plates, brain heart infusion broth, and Todd-Hewitt broth (THB) were obtained from BD Biosciences. Tryptic soy broth (TSB), tryptic soy agar, and hemin were obtained from MP Biomedicals. β-Nicotinamide adenine dinucleotide hydrate, gentamicin, and DMEM for the biofilm assay were obtained from Sigma-Aldrich. Phloretin was obtained from Cayman Chemicals. Crystal violet stain was obtained from Fisher Scientific. RAW 264.7 and NCI-H292 cells were obtained from ATCC. Normal and COPD human bronchial epithelial (HBE) cells were obtained from MatTek.

### Bacterial Strains and Exposure Conditions

NTHi and *M. catarrhalis* bacterial strains were grown overnight on chocolate agar plates and *S. pneumoniae* was grown on sheep blood agar plates (37°C, 15h, 5% CO_2_). PAO1 was grown in TSB (37°C, 15h, with shaking). A portion was subcultured in fresh TSB (37°C, 2h, with shaking), centrifuged (4000 RCF, 5 min), and washed in PBS. Bacterial concentrations were determined by optical density (OD_600_) and were confirmed by serial dilutions on the appropriate agar plates. A detailed table of bacterial growth conditions for each assay is provided (**Supplemental Table 2**).

### Phloretin Antibacterial Activity Assay

The antibacterial activity of phloretin was examined in a cell-free system by incubating multiple doses of phloretin with bacteria in 96-well plates. Vehicle, phloretin (0.1-1 mM, diluted in PBS), or gentamicin (100 µg/mL) was incubated with control (appropriate bacterial diluent), NTHi (10^6^ colony forming units (CFU), in brain heart infusion broth supplemented with hemin and β-nicotinamide adenine dinucleotide hydrate (sBHI)), *M. catarrhalis* (10^5^ CFU, in THB), *S. pneumoniae* (5x10^5^ CFU, in THB supplemented with yeast extract), or PAO1 (10^5^ CFU, in 20% TSB) at a 1:1 v/v. Microtitre plate lids were coated with 0.5% Triton X-100 in ethanol to prevent condensation. Plates were incubated (37°C, 18 h) and the OD_570_ was determined hourly using a plate reader (BioTek). Prior to a reading, the plate was shaken for 10 min by the plate reader. Following the assays, the bacteria mixtures were serially diluted and plated on their corresponding agar plates to manually count the CFU. Two to three independent trials were conducted with each condition performed in triplicate for each assay (*n*=6-9 total wells per condition).

### Crystal Violet Biofilm Assay

Quantification of crystal violet stained biofilm was determined using a microtiter plate assay, as modified by O’Toole (O’Toole, 2011). Vehicle (0.1% DMSO), 100 µM phloretin, or 100 µg/mL gentamicin in PBS was added to control (PBS), NTHi (10^6^ CFU in sBHI), *M. catarrhalis* (10^6^ CFU in THB), *S. pneumoniae* (10^5^ CFU in THB), or PAO1 (10^6^ CFU in DMEM) at a 1:1 v/v in 96-well plates. Plates were incubated under the same conditions (37°C, 16 h, 5% CO_2_) except for PAO1, which was incubated without CO_2_. Cells were stained with 0.5% crystal violet (22°C, 20 min with shaking). Plates were dried (22°C, 16 h) and bound dye was released from the cells with methanol and quantified by measuring the OD_570_. Two independent trials were conducted with each condition performed in triplicate for each assay (*n*=6 total per condition).

### Cell Culture

NCI-H292 cells (a human pulmonary epithelial cell line) or RAW 264.7 cells (a mouse macrophage cell line) were grown in 75 cm^2^ tissue culture flasks in RPMI-1640 (ATCC) or DMEM (Thermo Fisher Scientific), respectively, supplemented with 10% FBS and penicillin (100 U/mL) and streptomycin (100 µg/mL). HBE cells derived from normal and COPD donors were seeded onto collagen-coated T75 flasks and grown in bronchial epithelial growth medium (Lifeline Cell Technology). For experiments, cells were seeded into 12-well plates (NCI-H292 cells: 5,000 cells/cm^2^; HBE cells: 5,000 cells/cm^2^), or 96-well plates (RAW 264.7 cells: 312,500/cm^2^). Upon confluency, medium was replaced with supplement-free medium prior to experiments (18h). All cultures were maintained at 37°C in 5% CO_2_/95% air.

### Antibacterial Co-Culture Assay

The antibacterial activity of phloretin against NTHi was measured in the presence of NCI-H292 cells using an assay adapted from previous studies (Morey et al., 2011; Euba et al., 2017). Cells were pretreated for 1 h with 0.1 mM phloretin and exposed to NTHi (3x10^4^ CFU/mL, 500 µL) diluted in 20% sBHI in RPMI. After 4 h, the cells were washed twice with PBS and lysed with 0.1% w/v saponin (Alfa Aesar); this was considered the adherent and intracellular collection. Another collection of treated cells were washed twice with PBS and treated with 200 µg/mL gentamicin (1 h) prior to lysing; this was considered the intracellular collection. Two independent trials were conducted with biological replicates performed in triplicate for each assay (*n*=6 total). Each replicate was serially diluted and plated on chocolate agar and incubated overnight (37°C, 15 h, 5% CO_2_). The CFU was enumerated to compare the number of bacteria in each collection.

### ELISA

The anti-inflammatory activity of phloretin against the COPD pathogens in RAW 264.7 cells was measured. Cells were pretreated 1 h with vehicle (0.1% DMSO) or 0.1 mM phloretin and exposed to NTHi (2x10^5^ CFU/mL, 50 µL), *M. catarrhalis* (2x10^5^ CFU/mL, 50 µL), *S. pneumoniae* (2x10^5^ CFU/mL, 50 µL), or PAO1 (2x10^4^ CFU/mL, 50 µL) for 2h. Tumor necrosis factor (TNF) was measured in the supernatant of cells by ELISA (BioLegend). Two independent trials were conducted with biological replicates performed in triplicate (*n*=6 total) and 2 technical replicates for the ELISA.

### Real-Time Quantitative PCR

Normal and COPD HBE cells were pretreated with 0.1 mM phloretin (1 h) and exposed to 500 µL 1.5x10^7^ CFU/mL of NTHi. RNA was extracted from HBE cells after 2 h with TRI Reagent (Sigma-Aldrich) and reverse transcribed into first-strand cDNA (Bio-Rad). RT-qPCR was performed using TaqMan gene expression master mix (Life Technologies) and primers with a thermal cycler (Applied Biosystems) under the following conditions: 50°C (2 min), 95°C (10 min), and 40 cycles of 95°C (15 sec) and 60°C (1 min). Interleukin (IL) 6, IL8, C-C motif chemokine ligand 20 (CCL20), and ribosomal protein (RPL32) transcripts were analyzed. Relative gene expression was calculated using the 2^-ΔΔ^CT method normalized to RPL32. Two independent trials were conducted with biological replicates performed in triplicate (*n*=6 total), each with 3 technical replicates for the RT-qPCR assays.

### Cytotoxicity Assessment

RAW 264.7 cells and HBE cells were pretreated with vehicle (0.1% DMSO) or 0.1 mM phloretin (1 h) and exposed to medium control or bacteria. Lactate dehydrogenase activity was measured using CytoTox-ONE reagent (Promega). Two independent trials were conducted with biological replicates performed in triplicate (*n*=6 total), with no technical replicates.

### Animal Experiments

Animal studies were conducted with approval of the Institutional Animal Use and Care Committee of the University of Pittsburgh. FVB/NJ mice (Jackson Laboratories, female and male, 8 wk) were housed under pathogen-free conditions and maintained on an AIN-93G diet (Envigo) for 3 days prior to the start of the experiment. Mice were then fed an AIN-93G standard diet without or with the addition of 0.157% phloretin *ad libitum* for 1 week. Diet was administered within 2 weeks upon receipt from the manufacturer to maintain freshness and efficacy. Single colonies of NTHi were incubated in sBHI (37°C, 1.5 h, with shaking). The culture was centrifuged (4000 RCF), washed twice in PBS, and adjusted to an OD_600_ of 2. Mice were inoculated intratracheally with PBS or 10^5^ CFU of NTHi. The dose was confirmed by plating serial dilutions on chocolate agar plates. After 24 h, mice were anesthetized with sodium pentobarbital. The dorsal aorta was severed and the diaphragm punctured. Bronchoalveolar lavage (BAL) fluid was collected by cannulating the trachea and washing the lungs with 1 mL of PBS, reinserted twice.

To analyze CFU content, lungs were harvested and homogenized in 500 µL PBS. Serial dilutions of the homogenate and BAL fluid were plated on chocolate agar and CFU was counted. Results presented are total CFU in the lung homogenate. BAL was also analyzed for chemokine (C-X-C motif) ligand 1 (CXCL1 aka GRO-1) protein by a Luminex-based assay (Research And Diagnostic Systems).

### Statistical Analysis

All cell-free and *in vitro* data is expressed as mean ± SEM of 2-3 independent trials, with every condition performed in triplicate within each trial. *In vivo* data is expressed as mean ± SEM of 2 independent trials, with 5-6 mice tested within each trial. Dot plots represent results from the combined trials, with each biological replicate presented as a single dot. Data was analyzed using GraphPad software and significant difference (*P*<0.05) between groups was assessed by t-test or analysis of variance (ANOVA) with multiple comparisons test (Dunnett’s, Tukey’s).

## Results

### Phloretin Inhibits Bacterial Growth and Biofilm Formation

**Figure 1:**
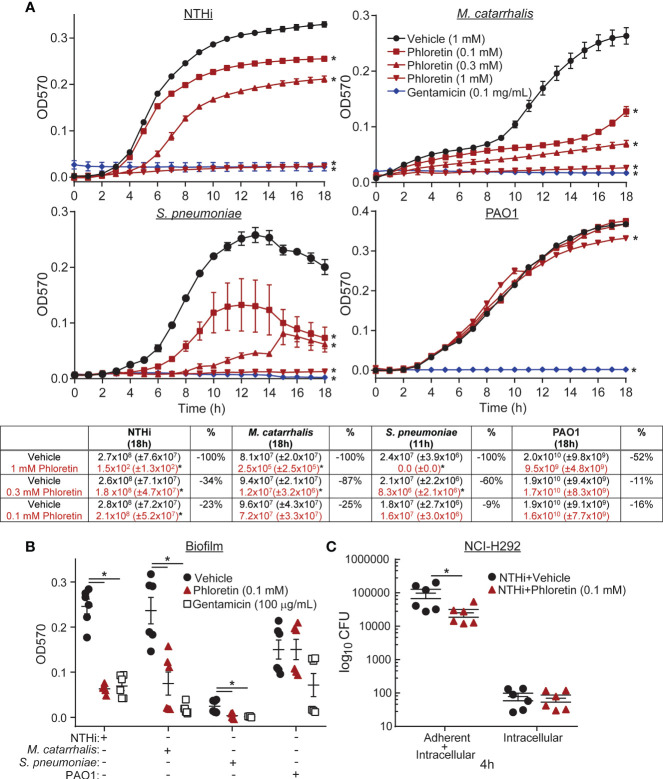
Phloretin inhibits COPD pathogen growth and biofilm formation in abiotic assays and in coculture with lung epithelial cells. **(A)** Phloretin delays COPD pathogen growth in a dose dependent manner. (Graphs) 10^6^ CFU nontypeable *Haemophilus influenzae* (NTHi), 10^5^ CFU *Moraxella catarrhalis* (*M. catarrhalis*), 5x10^5^ CFU *Streptococcus pneumoniae* (*S. pneumoniae*), and 10^5^ CFU *Pseudomonas aeruginosa* strain PAO1 were grown with vehicle, phloretin (0.1-1 mM), or gentamicin (100 µg/mL) at a 1:1 v/v over 18h. Bacterial growth was assessed by measuring the OD_570_ hourly. *Significantly different from vehicle+pathogen treated cells (*P*<0.05) assessed by two-way ANOVA followed by Dunnett’s multiple comparisons test. (Table) Microtitre results were confirmed by plating the treated bacteria from the assays on corresponding agar plates and manually counting the CFU. The percent change (%) in the antibacterial effect of phloretin relative to vehicle is presented. Results are the means ± SEM of 2-3 independent trials with 3 replicates per condition tested within each trial (*n*=6-9). *Significantly different from vehicle+pathogen treated cells (*P*<0.05) assessed by paired t-test. **(B)** Phloretin inhibits COPD pathogen biofilm formation on abiotic plastic surfaces. 10^6^ CFU NTHi, 10^6^ CFU *M. catarrhalis*, 10^5^ CFU *S. pneumoniae*, and 10^6^ CFU PAO1 were grown with vehicle, phloretin (0.1 mM), or gentamicin (100 µg/mL) at a 1:1 v/v. Biofilm biomass was quantified by crystal violet staining (OD_570_). Results are the means ± SEM of 2-3 independent trials with 3 replicates per condition tested within each trial (*n*=6-9). *Significantly different from vehicle+pathogen treated cells (*P*<0.05) as determined by one-way ANOVA followed by Dunnett's multiple comparisons test. **(C)** Phloretin reduces NTHi adhesion to NCI-H292 cells. NCI-H292 cells were pretreated with vehicle or 0.1 mM phloretin and exposed to 500 µL of 3x10^4^ CFU/mL NTHi (4 h, 37°C, 5% CO_2_). Cells were lysed to collect adherent and intracellular bacteria ("adherent+intracellular") or treated with gentamicin and then lysed to collect intracellular bacteria ("intracellular" collection). Bacteria were enumerated in each collection by plating serial dilutions on chocolate agar plates and counting the CFU. Results are the means ± SEM of 2 independent trials with 3 biological replicates per condition tested within each trial (*n*=6). *Significantly different from vehicle+NTHi treated cells (*P*<0.05) as assessed by paired t-test.

A microtiter plate-based antibacterial assay was used to examine the antibacterial capability of phloretin against the COPD pathogens. Bacteria were incubated with vehicle, 0.1-1 mM phloretin, or 100 µg/mL gentamicin (antibiotic control). Phloretin inhibited NTHi, *M. catarrhalis*, *S. pneumoniae*, and PAO1 growth (**Figure 1A**). Inhibition was significant at ≥ 0.1 mM for NTHi, *M. catarrhalis*, and *S. pneumoniae*, and ≥ 1 mM for PAO1. Microtitre results were confirmed by manual CFU counts of the wells following the completion of the assays (18 h) and were comparable to the growth curves, where phloretin had antibacterial activity against all of the bacterial strains in a dose-dependent manner, except for *S. pneumoniae* (**Supplemental Table 3**). This may be due to a loss of viability of the bacteria. When the CFU was manually enumerated at the beginning of the stationary phase (11 h), phloretin (0.3-1 mM) displayed antibacterial activity against *S. pneumoniae* (**Figure 1A**). In another abiotic assay, biofilm biomass was measured by crystal violet staining (OD_570_). Phloretin (0.1 mM) inhibited biofilm formation of NTHi, *M. catarrhalis*, and *S. pneumoniae* but not PAO1 (**Figure 1B**).

The antibacterial effect of phloretin was examined in co-culture with NCI-H292 cells, a human pulmonary epithelial cell line. Over time, phloretin inhibited NTHi adhesion to the cells over 8 h. After 4 h exposure, phloretin reduced adherent bacteria but did not have an impact on bacterial invasion at this time (**Figure 1C**). Phloretin was previously found to non-cytotoxic to NCI-H292 cells at this dose, as measured by lactate dehydrogenase release and ATP levels (Birru et al., 2020).

### Phloretin Reduces Inflammatory Cell Recruitment Induced by COPD Associated Pathogens

**Figure 2:**
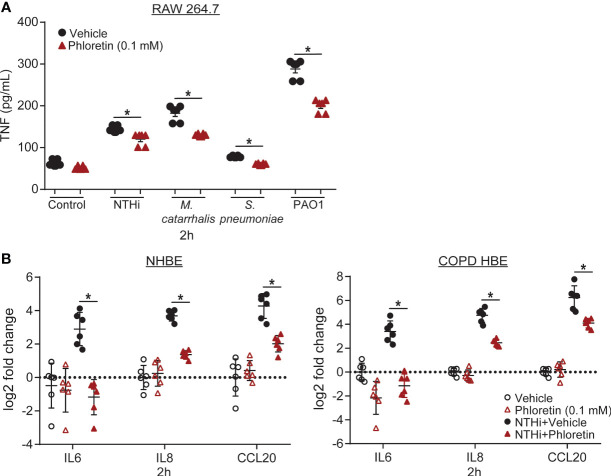
Phloretin exhibits anti-inflammatory activity in COPD pathogen-induced RAW 264.7 cells and nontypeable *Haemophilus influenzae* (NTHi)-induced human bronchial epithelial (HBE) cells. **(A)** COPD pathogens significantly increase TNF secretion from RAW 264.7 cells, which phloretin inhibits. RAW 264.7 cells were pretreated with 0.1 mM phloretin (1 h) and exposed to medium control, NTHi (2x10^5^ CFU/mL, 50 µL), *Moraxella catarrhalis (M. catarrhalis)* (2x10^5^ CFU/mL, 50 µL), *Streptococcus pneumoniae (S. pneumoniae)* (2x10^5^ CFU/mL, 50 µL), or PAO1 (2x10^4^ CFU/mL, 50 µL) for 2h. TNF was measured in the supernatant by ELISA. Results are the mean ± SEM of 2 independent trials with 3 biological replicates per condition tested within each trial (*n*=6). *P* values were assessed by one-way ANOVA followed by Tukey’s multiple comparisons test to compare vehicle to vehicle+pathogen treated cells and to compare vehicle to phloretin treated cells for each condition. *Significantly different from vehicle treated cells (*P*<0.05). **(B)** Phloretin inhibits NTHi-induced inflammatory cytokine production in normal and COPD HBE cells. Normal and COPD HBE cells were pretreated with 0.1 mM phloretin (1 h) and exposed to 500 µL 1.5x10^7^ CFU/mL of NTHi (2 h). Interleukin (IL6), IL8, C-C motif chemokine ligand 20 (CCL20), and ribosomal protein L32 (RPL32) transcripts were measured by RT-qPCR. Values are log2 fold change of IL6, IL8, and CCL20 mRNA normalized to RPL32 relative to vehicle control treated cells. Results are the means ± SEM of 2 independent trials with 3 biological replicates per condition tested within each trial (*n*=6). *P* values were assessed by one-way ANOVA followed by Tukey’s multiple comparisons test to compare vehicle to vehicle+NTHi treated cells and to compare vehicle to phloretin treated cells for each transcript. *Significantly different from vehicle treated cells (*P*<0.05).

LPS is a component of the cell wall of gram-negative bacteria that is recognized and bound by Toll-like receptors (TLRs) on cell surfaces, initiating an innate immune response (Lee and Kim, 2007; Knobloch et al., 2011). NTHi contains short-chain LPS, or lipooligosaccharide (LOS), with structural variant oligosaccharide components attached to its core region (Choi et al., 2014) and can bind to TLR4 in lung epithelial cells (Birru et al., 2020) and macrophages (Lorenz et al., 2005). *S. pneumoniae* is a gram-positive bacterium that does not express LPS. However, TLR2 and TLR4 recognize lipoteichoic acid, present on its cell wall, and pneumolysin, which it secretes (Sánchez-Tarjuelo et al., 2020). Upon TLR activation, macrophages secrete cytokines to stimulate activation and migration of neutrophils and other immune cells to the site of injury (Wu et al., 2009). To evaluate the anti-inflammatory effects of phloretin, we exposed RAW 264.7 macrophages to the COPD associated pathogens and measured TNF secretion as a marker of inflammation. TNF protein released from the cells into the supernatant was measured at 2 h, prior to the phloretin-induced inhibition of bacterial growth (**Figure 1**). Each pathogen increased TNF secretion relative to vehicle control (**Figure 2A**). Phloretin pretreatment inhibited pathogen-induced TNF secretion in RAW 264.7 cells, indicating its anti-inflammatory role. In pathogen exposed cells at 24 h, vehicle treated cells had a significant increase in TNF secretion while levels in phloretin treated cells remained at baseline and were comparable to the vehicle plus control treatment group (data not shown). This may be a result of both the anti-inflammatory and antimicrobial activity of phloretin. At this concentration, phloretin did not induce cytotoxicity in RAW 264.7 cells (**Supplemental Figure 2A**). Furthermore, the bacteria did not impact cell viability (**Supplemental Figure 2A**).

### Phloretin Maintains Anti-Inflammatory Activity in a COPD Model of Infection

To confirm these results in a human model of infection, we used HBE cells derived from normal and COPD subjects. Airway epithelial cells release cytokines in response to pathogens to induce chemotaxis of inflammatory mediators to the site of injury. In HBE cells, we measured IL6, an inflammatory mediator, IL8, a neutrophil chemoattractant, and CCL20, a lymphocyte chemoattractant, which have all been found to be induced by respiratory epithelial cells following NTHi exposure (Clemans et al., 2000; Amatngalim et al., 2017). NTHi increased IL6, IL8, and CCL20 transcript levels in HBE cells. IL6 and CCL20 transcript levels were significantly higher in COPD HBE cells compared to NHBE cells. Phloretin suppressed these biomarkers in both NHBE and COPD HBE cells (**Figure 2B**). At this concentration, phloretin did not induce cytotoxicity in HBE cells (**Supplemental Figure 2B**). NTHi had a small but significant effect on cell viability in NHBE cells but not COPD HBE cells (**Supplemental Figure 2B**).

### A Diet Supplemented With Phloretin Inhibits NTHi-Induced Bacterial Growth and Inflammation in Mouse Lung

**Figure 3:**
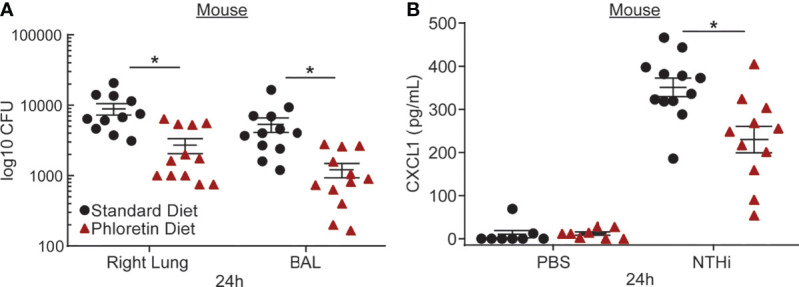
Phloretin reduces nontypeable *Haemophilus influenzae* (NTHi) burden and inflammation in mouse lungs. **(A)** Phloretin inhibits NTHi growth in mouse lungs. FVB/NJ mice (8 wks) were supplied with food pellets with or without phloretin supplementation ad libitum (0.157%, 1 wk) and exposed to NTHi (10^5^ CFU, 24 h) intratracheally. Bacterial counts were measured in lung homogenate and BAL at 24 h. Results are the means ± SEM of 2 independent trials of 5-6 mice per condition tested within each trial (*n*=11-12). *Significantly different from standard diet exposed mice (*P*<0.05) as determined by unpaired t-test. **(B)** Phloretin inhibits C-X-C motif chemokine ligand 1 (CXCL1) protein, a neutrophil chemoattractant, induced by NTHi in BAL fluid. CXCL1 was measured in BAL fluid with a Luminex assay. Results are means ± SEM (*n*=8-12). *P* values were assessed by one-way ANOVA followed by Tukey’s multiple comparisons test to compare standard diet+PBS to standard diet+NTHi treated mice and to compare standard diet to phloretin diet treated mice. *Significantly different from standard diet treated mice (*P*<0.05).

To examine its antibacterial effects *in vivo*, mice were supplied a diet supplemented with phloretin (0.157%, 1 wk) and exposed intratracheally to NTHi (24 h). Serial dilutions of the bronchoalveolar lavage fluid and lung tissue homogenate were plated to quantify the bacterial load by enumerating the CFU. NTHi CFU was reduced in the BAL fluid and lung tissue of mice exposed to phloretin (**Figure 3A**). Previously, we showed that NTHi exposure induced neutrophil infiltration into the lungs, which was inhibited by phloretin treatment (Birru et al., 2020). To strengthen these results, we measured CXCL1, a neutrophil chemoattractant, in BAL fluid from NTHi-treated mice. CXCL1 increased after NTHi exposure and was reduced with a phloretin diet (**Figure 3B**). The reduction in neutrophils and CXCL1 in mouse lung was in agreement with the inhibition of IL8 in HBE cells. Together these findings indicate that phloretin displays both bacteriostatic and anti-inflammatory activity against COPD associated pathogens *in vitro* and *in vivo*.

## Discussion

Pathophysiological changes in COPD include excessive mucus production and impaired mucociliary clearance. These changes contribute to a shift in the respiratory microbiome, increasing the colonization and growth of pathogenic microbiota (Sze et al., 2014). Dietary polyphenols have been proposed to improve COPD symptoms due to their broad antioxidant capacity, and have been shown to improve lung function (measured by forced expiratory volume in one second) and arterial oxygen tension (measured by PaO2) (Domej et al., 2014). In this study, we also demonstrate that phloretin, an apple polyphenol, may provide antibacterial and anti-inflammatory activity in the context of bacterial infections most common in COPD. In practice, consumption of phloretin as a supplement would be a more reasonable to achieve the doses used in our experiments rather than increased apple intake. For example, the mouse dose used in our experiments is equivalent to approximately 200 apples per day (Birru et al., 2020).

In a cell-free system, we found that phloretin demonstrated bacteriostatic and anti-biofilm activity against the COPD pathogens NTHi, *M. catarrhalis*, *S. pneumoniae*, and to a lesser extent, PAO1. Gentamicin similarly imparted less antibacterial activity against PAO1 relative to the other bacteria. Compared to other bacteria, PAO1 is particularly resistant to antibiotics due to multidrug efflux transporters, low outer membrane permeability, and production of antibiotic deactivating enzymes (Poole, 2001; Pang et al., 2019). Therefore, antimicrobial agents, such as phloretin and gentamicin, may be less effective against this bacteria.

Using NCI-H292 as a coculture model of infection, we found that phloretin modulated NTHi adhesion. We also demonstrated that NTHi clearance is enhanced in mice supplied a diet supplemented with phloretin, as measured in lung tissue homogenate and BAL fluid. Currently, the primary pharmacological intervention for treating bacterial-induced exacerbations is antibiotic therapy, which may reduce an individual’s frequency of exacerbations (Ramos et al., 2014; Huckle et al., 2018) but overall, may increase the risk of resistant bacterial growth in patients (Huckle et al., 2018). Polyphenols reduce bacterial antibiotic resistance mechanisms, such as drug efflux, and potentiate antibiotic efficacy (Dey et al., 2015; Suriyanarayanan and Sarojini Santhosh, 2015), including compounds in the chalcone structural family in which phloretin belongs (Ferraz et al., 2020; Hellewell and Bhakta, 2020). These results provide the basis for further studies to examine the ability of phloretin to improve bacterial-induced symptoms in COPD or to augment antibiotic therapy and potentiate its efficacy.

Pathogens induce respiratory neutrophil-driven inflammation in persons with COPD, contributing to airway epithelial cell damage and AECOPD (Sethi and Murphy, 2001). We demonstrated that phloretin reduced the pathogen-induced increase in chemokine transcripts in NHBE and COPD HBE cells. These chemokines can amplify inflammation by recruiting neutrophils and lymphocytes to the lung, activating the adaptive immune system to respond to the injury. In mouse lung, NTHi stimulated inflammation, particularly neutrophil influx as measured by CXCL1, which was suppressed by a phloretin diet. Previously, phloretin was found to inhibit IL8/CXCL1 *in vitro* in LPS/IFNγ exposed human monocytic cells (Jung et al., 2009) and TNF-exposed human keratinocyte cells (Huang et al., 2015). However, the antibacterial effect of the phloretin diet may have reduced the initial inflammatory response to NTHi, therefore also contributing to the CXCL1 inhibition. Airway neutrophilia is a common feature among all subtypes of COPD and contributes to key pathological features of the disease, including airway obstruction, lung function decline, and emphysema (Birru and Di, 2012; Jasper et al., 2019). Therefore the effectiveness of phloretin in reducing bacterial-induced neutrophil mediated inflammation is promising for a therapeutic intervention.

Pathogenic bacteria in COPD is associated with disease morbidity, lung function decline, and AECOPD (Sethi and Murphy, 2001). Our studies demonstrate the antibacterial and anti-inflammatory effects of phloretin in acute models of infection using COPD associated bacteria. Taken together, our results provide a basis for the potential usage of a phloretin isolate as a therapeutic agent against pathological features induced by bacterial infections in COPD. Future studies are warranted to determine the impact of phloretin in *in vitro* and pre-clinical mouse models of COPD.

## Data Availability Statement

The data that support the findings of this study are available from the corresponding author upon reasonable request.

## Ethics Statement

The animal study was reviewed and approved by Institutional Animal Use and Care Committee of the University of Pittsburgh.

## Author Contributions

All authors contributed to the manuscript and approved the submitted version.

## Conflict of Interest

The authors declare that the research was conducted in the absence of any commercial or financial relationships that could be construed as a potential conflict of interest.

## Publisher’s Note

All claims expressed in this article are solely those of the authors and do not necessarily represent those of their affiliated organizations, or those of the publisher, the editors and the reviewers. Any product that may be evaluated in this article, or claim that may be made by its manufacturer, is not guaranteed or endorsed by the publisher.

## Abbreviations

AECOPD, acute exacerbations of chronic obstructive pulmonary disease; CXCL1, chemokine (C-X-C motif) ligand 1; CFU, colony forming unit; COPD, chronic obstructive pulmonary disease; HBE, human bronchial epithelial; IL, interleukin; LPS, lipopolysaccharide; *M. catarrhalis*, *Moraxella catarrhalis*; NTHi, nontypeable *Haemophilus influenzae*; *P. aeruginosa*, *Pseudomonas aeruginosa*; sBHI, brain heart infusion broth supplemented with hemin and β-nicotinamide adenine dinucleotide hydrate; *S. pneumoniae*, *Streptococcus pneumoniae*; THB, Todd-Hewitt broth; TLR, Toll-like receptor; TNF, tumor necrosis factor; TSB, tryptic soy broth.
