# Supplementary material for: Antimicrobial and Anti-Inflammatory Activity of Apple Polyphenol Phloretin on Respiratory Pathogens Associated With Chronic Obstructive Pulmonary Disease
Source: Front Cell Infect Microbiol. 2021 Nov 22;11:652944. doi: 10.3389/fcimb.2021.652944 (PMC8645934; doi:10.3389/fcimb.2021.652944)
Supplement: Supplementary file 1 [file DataSheet_1.pdf]

Antimicrobial and anti-inflammatory activity of apple polyphenol phloretin on respiratory pathogens

Rahel L. Birru, Kiflai Bein, Natalya Bondarchuk, Heather Wells, Qiao Lin, Y. Peter Di, George D. Leikauf

Supporting Information

SUPPLEMENTAL TABLE 1

Category	Name	Supplier	Catalog #	Comments
Reagents	Phloretin	Cayman Chemicals (Ann Arbor, MI)	14452	Reconstituted in DMSO
Cell Culture	75cm² Cell Culture Flask	Corning Costar (Tewksbury, MA)	3276	
	96 well plates	Greiner Bio-One (Austria)	655180	
	12 well plates	Corning Costar	3513	
	NCI-H292 cells	ATCC (Manassas, VA)	CRL-1848	
	RAW 264.7 cells	ATCC	TIB-71	
	Normal Human Bronchial Epithelial (HBE) cells	MatTek (Ashland, Massachusetts)	NHBE-CRY	Cultured on PureCol collagen-coated plates
	COPD Human Bronchial Epithelial cells	MatTek	NHBE-CRY-DS	Cultured on PureCol collagen-coated plates
	Fetal Bovine Serum	Thermo Fisher Scientific (Waltham, MA)	26140	
	Penicillin Streptomycin 100X Solution	GE Healthcare Life Sciences (Marlborough, MA)	SV30010	
	RPMI-1640	ATCC	30-2001	For NCI-H292 cell culture
	Dulbecco’s Modified Eagle’s Medium (DMEM)	Thermo Fisher Scientific	11995-065	For RAW 264.7 culture
	Bronchial epithelial growth medium (BEGM)	Lifeline Cell Technology (Oceanside, CA)	LL-0023	For HBE cell culture

Category	Name	Supplier	Catalog #	Comments
Cell Culture	PureCol	Advanced BioMatrix	5005	PureCol was diluted 100 µg/mL (1:30) in water and incubated on plates/flasks for 2h (room temperature). Excess solution was removed and flasks/plates were dried overnight in a cell culture hood. Plates were exposed to UV light for 30 min and rinsed with BEGM before culturing cells.
Bacteria	Nontypeable *Haemophilus influenzae* (NTHi)	Provided by Dr. Hong Wei Chu, National Jewish Health	Clinical isolate	Strain 12, from middle ear infection
	Moraxella catarrhalis	ATCC	25238	
	Streptococcus pneumoniae	ATCC	6303	
	Pseudomonas aeruginosa	ATCC	BAA-47	
	Brain Heart Infusion broth (BHI)	BD Biosciences (San Jose, CA)	237500	
	β-Nicotinamide adenine dinucleotide hydrate (NAD)	Sigma-Aldrich	N1511	-Added 100 mg NAD to 10 mL distilled water and filtered sterilized<br>-For sBHI, added 100 µL NAD preparation to 10 mL BHI
	Hemin from Porcine	MP Biomedicals (Solon, OH)	198820	-Added 50 mg hemin to 5 mL 0.1 N NaOH, 5 mL ethylene glycol, 10 mL tris borate buffer, and 30 mL distilled water and filter sterilized<br>-For sBHI, added 10 µL of hemin preparation to 10 mL BHI
	Todd-Hewitt Broth (THB)	BD Biosciences	249240	
	Yeast Extract	Fisher Scientific (Hampton, NH)	BP9727	For THY broth, added yeast extract to THB for a final concentration of 0.2%
	Tryptic Soy Broth	MP Biomedicals	1010717	

Category	Name	Supplier	Catalog #	Comments
Bacteria	Chocolate II Agar (GC II Agar with Hemoglobin and IsoVitaleX)	BD Biosciences	B21267X	
	Trypticase Soy Agar (TSA II) with Sheep Blood	BD Biosciences	B21239X	
	Tryptic Soy Agar (TSA)	MP Biomedicals	1010617	
	PBS	Thermo Fisher Scientific	10010023	
Bacterial Growth and Biofilm Assays	96-Well Polystyrene Plates	Fisher Scientific	12-565-501	Used for bactericidal assays (plates were sterilized before use by UV light)
	Flat Bottom Cell Culture Plates	Corning Costar	3595	Used for biofilm assays
	Crystal Violet	Fisher Scientific	C581	Prepared 0.5% (w/v) solution: 0.125 g crystal violet powder to 5 mL methanol to solubilize and added 20 mL distilled H_2O
	DMEM - high glucose	Sigma-Aldrich	D5671	For PAO1 biofilm assay
	Gentamicin solution	Sigma-Aldrich	G1272	
	Saponin permeating solution, 0.5% w/v soln. in PBS (5X)	Alfa Aesar (Haverhill, MA)	J63209	Diluted to 1X in PBS
	Synergy 2 Plate Reader	BioTek (Winooski, VT)	7131000	
ELISA	LPS from Pseudomonas aeruginosa 10	Sigma-Aldrich	L8643	
	Mouse TNF-α ELISA MAX Deluxe	BioLegend (San Diego, CA)	430904	
	Stop Solution for TMB Substrate	BioLegend	423001	
RT-qPCR	TRI Reagent	Sigma-Aldrich	93289	
	iScript cDNA Synthesis Kit	Bio-Rad (Hercules, CA)	1708891	
	TaqMan gene expression master mix	Life Technologies (Carlsbad, CA)	4369016	

Category	Name	Supplier	Catalog #	Comments
RT-qPCR	TaqMan Gene Expression Assay: RPL32	Life Technologies	Hs00851655_g1	
	TaqMan Gene Expression Assay: IL6	Life Technologies	Hs00174131_m1	
	TaqMan Gene Expression Assay: CCL20	Life Technologies	Hs00355476_m1	
	TaqMan Gene Expression Assay: IL8	Life Technologies	Hs00174103_m1	
	ABI 7900HT	Applied Biosystems (Foster City, CA)	4351405	
Cytotoxicity Assay	CytoTox-ONE Homogeneous Membrane Integrity Assay	Promega (Madison, WI)	G7890	
Animal Trials	FVB/NJ mice	Jackson Laboratory (Bar Harbor, ME)	001800	
	Rodent Diet	Envigo (Huntingdon, United Kingdom)	Standard: TD.94045, 0.157% phloretin: TD.190074	AIN-93G purified diet was ordered with and without phloretin. Diet was administered within 2 weeks of delivery.
	Euthasol	Virbac AH, Incorporated (Fort Worth, TX)	200-071	
	Mouse Magnetic Luminex Assay	Research And Diagnostic Systems (Minneapolis, MN)	LXSAMSM	CXCL1 was measured in BAL fluid
	Bio-Plex 200 System	Bio-Rad		
Software	SigmaPlot 11	Systat Software (San Jose, CA)	Not applicable	
	GraphPad Prism 8	GraphPad Software, Inc. (La Jolla, CA)	Not applicable	

SUPPLEMENTAL TABLE 2: Growth Conditions of Bacteria

	Growth Conditions	Medium for Microtitre Antibacterial Growth Assays (Figure 1A)	Medium for Biofilm Assays (Figure 1B)	Medium for Cell Coculture Assays (Figure 1C, Figure 2)	Agar plates used to confirm experimental doses
**NTHi**	Chocolate agar<br>• 37°C, 5% CO_2 (15h)<br>• Single colonies added to indicated medium for subsequent experiments<br>• OD_{600} measured to adjust dose	BHI supplemented with hemin and NAD (sBHI)	sBHI	NCI-H292: 20% sBHI in RPMI;<br>RAW 264.7: DMEM;<br>HBE: BEBM	Chocolate agar
M. catarrhalis	Chocolate agar<br>• 37°C, 5% CO_2 (15h)<br>• Single colonies added to indicated medium for subsequent experiments<br>• OD_{600} measured to adjust dose	THB	THB	RAW 264.7: DMEM	Chocolate agar
S. pneumoniae	Sheep blood agar<br>• 37°C, 5% CO_2 (15h)<br>• Single colonies added to indicated medium for subsequent experiments<br>• OD_{600} measured to adjust dose	THB supplemented with 0.2% yeast (THY)	THB	RAW 264.7: DMEM	Sheep blood agar
***P. aeruginosa* (PAO1)**	TSB<br>• 37°C, shaking (15h)<br>• Subcultured in fresh TSB (37°C, shaking)<br>• Pelleted (4000 RCF, 5 min) and resuspended in indicated medium for subsequent experiments<br>• OD_{600} measured to adjust dose	20% TSB in PBS	DMEM	RAW 264.7: DMEM	Tryptic soy agar

Abbreviations: BEBM, Bronchial epithelial basal medium; BHI, brain heart infusion broth; *M. catarrhalis*, *Moraxella catarrhalis*; NAD, β-nicotinamide adenine dinucleotide hydrate; NTHi, nontypeable *Haemophilus influenzae*, *P. aeruginosa, Pseudomonas aeruginosa; S. pneumoniae, Streptococcus pneumoniae*; THB, Todd Hewitt broth; TSB, tryptic soy broth

SUPPLEMENTAL TABLE 3: Manual CFU Enumeration of *Streptococcus Pneumoniae* Microtitre Assay Growth Curve

	***S. pneumoniae* (18h)**	**%**
Vehicle<br>1 mM Phloretin	$3.5x10^3$ (±$1.7x10^3$)<br>0.0 (±0.0)	-100%
Vehicle<br>0.3 mM Phloretin	$4.9x10^3$ (±$2.6x10^3$)<br>$1.8x10^5$ (±$9.3x10^4$)	+3571%
Vehicle<br>0.1 mM Phloretin	$1.1x10^4$ (±$3.0x10^3$)<br>$1.1x10^4$ (±$3.0x10^3$)	+109%

Supplemental Table 3: **Confirmation of *Streptococcus pneumoniae* (*S. pneumoniae*) microtitre assays.** Microtitre results from the *S. pneumoniae* microtitre assays were confirmed by plating the treated bacteria from the assays (18 h) on sheep blood agar plates and manually counting the CFU. The percent change (%) in the antibacterial effect of phloretin relative to the vehicle control is presented. Results are the means ± SEM of 3 independent trials with 3 replicates per condition tested within each trial (n=9). P values were assessed by paired t-test.

SUPPLEMENTAL FIGURE 1

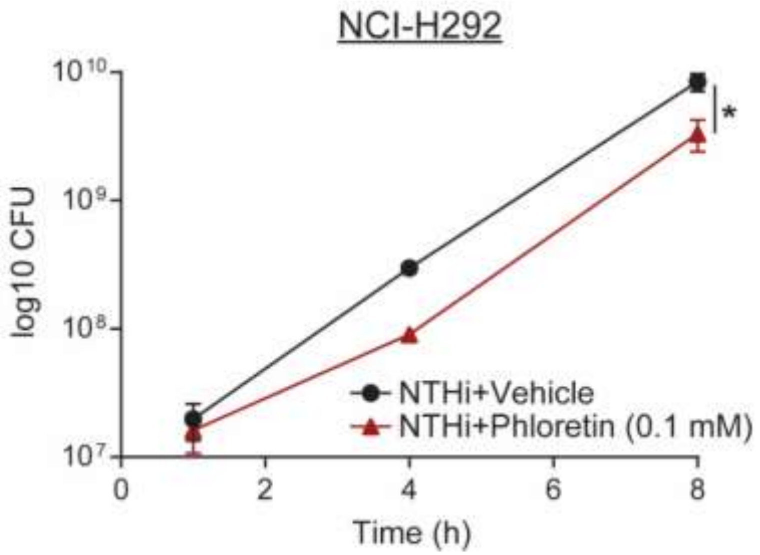

Supplemental Figure 1: Phloretin inhibits NTHi growth and adherence of NCI-H292 cells in a time-dependent manner. NCI-H292 cells were pretreated with vehicle or 0.1 mM phloretin and exposed to 500 µL of $3x10^4$ CFU/mL NTHi for 1, 4, and 8 h (37 °C, 5% CO_2). Cells were lysed to collect adherent bacteria. Bacteria were enumerated in each collection by plating dilutions on chocolate agar plates and counting the CFU. Data are the means ± SEM. Data are representative of 2 independent trials with 3 replicates per condition tested within each trial (*n*=6). The *P* value was assessed by two-way ANOVA.

SUPPLEMENTAL FIGURE 2

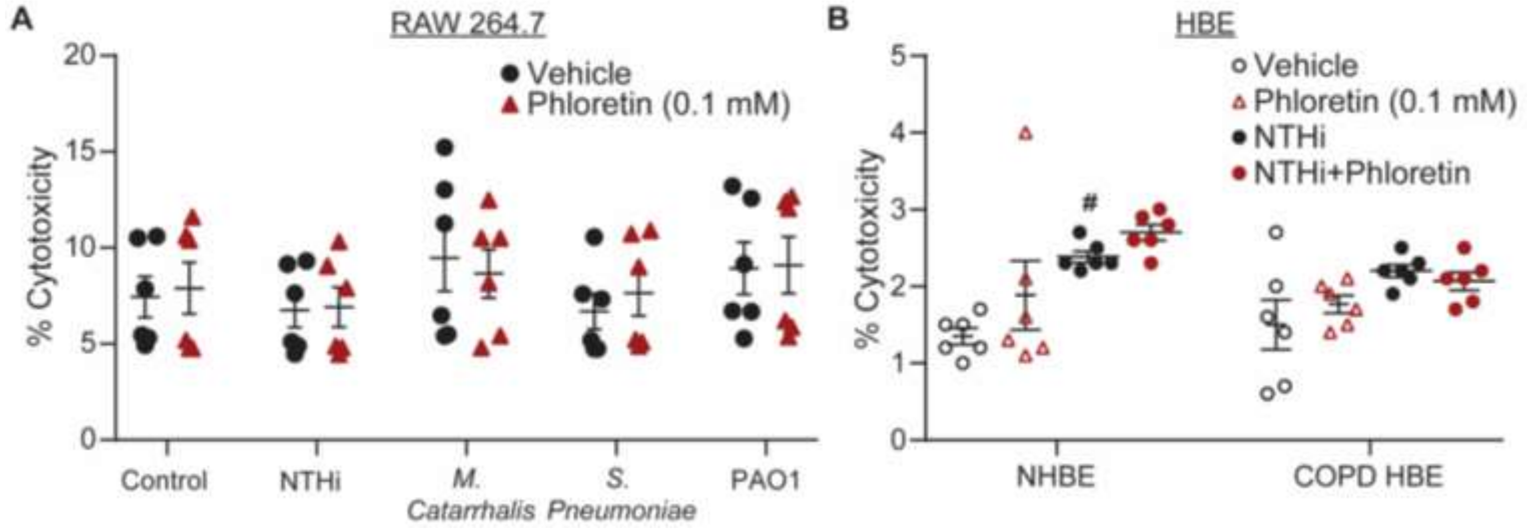

Supplemental Figure 2: Phloretin does not induce cytotoxicity in RAW 264.7 or human bronchial epithelial (HBE) cells. A. RAW 264.7 cells were pretreated with 0.1 mM phloretin (1 h) and exposed to medium control or NTHi ($2x10^5$ CFU/mL, 50 µL), *M. catarrhalis* ($2x10^5$ CFU, 50 µL), *S. pneumoniae* ($2x10^5$ CFU, 50 µL), or PAO1 ($2x10^5$, 50 µL). **B.** HBE cells were pretreated with 0.1 mM phloretin (1 h) and exposed to medium control or NTHi ($1.5x10^7$ CFU/mL, 500 µL). Cytotoxicity was determined by measuring lactate dehydrogenase release. Results are expressed as percent cytotoxicity relative to a maximum release lactate dehydrogenase control. Data are the means ± SEM. Data are representative of 2 independent trials with 3 replicates per condition tested within each trial (*n*=6). *P* values were assessed by one-way ANOVA followed by Tukey's multiple comparisons test to compare vehicle to vehicle+pathogen treated cells and to compare vehicle to phloretin treated cells for each condition. #Significantly different from vehicle+medium control treated cells ($P<0.05$).
